# Lapatinib promotes the incidence of hepatotoxicity by increasing chemotherapeutic agent accumulation in hepatocytes

**DOI:** 10.18632/oncotarget.3921

**Published:** 2015-04-23

**Authors:** ChunLing Dai, ShaoLin Ma, Fang Wang, HongYun Zhao, XingPing Wu, ZhenCong Huang, ZheSheng Chen, Kenneth To, LiWu Fu

**Affiliations:** ^1^ State Key Laboratory of Oncology in South China, Collaborative Innovation Center for Cancer Medicine, Cancer Center, Sun Yat-sen University, Guangzhou, China; ^2^ Department of Pharmaceutical Sciences, College of Pharmacy and Health Sciences, St. John's University, Queens, NY, USA; ^3^ School of Pharmacy, The Chinese University of Hong Kong, Hong Kong SAR

**Keywords:** lapatinib, ABC transporters, hepatotoxicity, pharmacokinetic

## Abstract

Lapatinib has been used in combination with capecitabine or paclitaxel to treat patients with progressive HER2-overexpressing metastatic breast cancer (MBC). Unfortunately, an increased incidence of hepatotoxicity had been reported in the combinational therapy. The aim of this study was to investigate the potential mechanisms of this combinational therapy. We found that the patients receiving lapatinib and paclitaxel treatment showed a higher incidence of hepatobiliary system disorders than those receiving paclitaxel alone. Lapatinib was shown to increase the accumulation of doxorubicin in ABCB1-overexpressing hepatocellular cancer cells and normal liver tissues without altering the protein level of ABCB1. Pharmacokinetic studies revealed that lapatinib could increase the systematic exposure of paclitaxel and doxorubicin. Moreover, the *in vivo* experiments showed that the levels of alanine aminotransferase and serious hepatocyte injury in the group of lapatinib plus chemotherapeutic agent were significantly higher than those in the group of single chemotherapeutic agent such as paclitaxel or doxorubicin. Our study thus revealed for the first time that the higher incidence of hepatotoxicity during this combinational treatment was due to the increased drug accumulation in hepatocytes mediated by the inhibition of ABCB1 by lapatinib. Appropriate dose adjustment may be needed to optimize the combination therapy.

## INTRODUCTION

ATP-binding cassette (ABC) transporters play a key role in normal pharmacology, xenobiotic protection, stem cell maintenance and is involved in the multidrug resistance (MDR) phenotype of human cancers [1]. They are efflux transporters that actively extrude a wide variety of chemically unrelated compounds including anthracyclines, vinca alkaloids, epipodophyllotoxins and tanxanes out of cells [2]. P-glycoprotein (ABCB1/MDR1), multidrug resistance-associated protein 1 (ABCC1/MRP1) and breast cancer resistance protein (ABCG2/MXR) are three major ABC transporters associated with MDR [1]. So far, ABCB1, ABCC1 and ABCG2 have been found overexpressed in many chemotherapeutic-resistant tumors such as colon cancer, liver cancer and kidney cancer [3]. With a crucial protective role, these ABC transporters are also expressed at high levels in intestinal epithelial cells, proximal renal tubular cells, placental trophoblast cells, the blood brain barrier, and on the biliary surface of hepatocytes [4, 5]. Under normal physiological condition, the transporters prevent the accumulation of xenobiotics and subsequently reducing toxic effect in various organs [6]. Previously, we showed that lapatinib inhibited the function of ABC transporters (including ABCB1, ABCC1 and ABCG2) and enhanced the anticancer activity of chemotherapeutic agents in ABC transporter-overexpressing cancer cells [7, 8]. In liver, ABCB1 is expressed at the apical side of bile canaliculus so that the transporter mediates the hepatic elimination of xenobiotics and drugs into the bile [9]. To this end, ABCB1 has been reported to protect again hepatotoxicity from anticancer drugs such as trabectedin [10]. It follows that the accumulation of conventional ABC substrate chemotherapeutic agents in liver may be increased when the patients were also concomitantly administered with an ABC transporter inhibitor.

Drug-induced hepatotoxicity is a major dose-limiting adverse effect hindering the clinical application of many drugs. The U.S. Food and Drug Administration (FDA) had delayed many drugs approval and withdrawn approved drugs from the market because of severe hepatotoxicity, such as bithionol cobalt salts, sulfathiazole and oxyphenisatin [11]. Liver is the major site of drug metabolism and detoxification. More than 80% of blood flow of liver come from the gastrointestinal tract and the liver has a high capacity for both phase I and phase II biotransformation [12]. Therefore, liver is vulnerable to the toxic effect from chemotherapeutic agents. The most catastrophic consequence is the occurrence of acute liver failure that leads to death or requires liver transplantation. In recent years, the combination chemotherapy is the mainstay treatment option for many cancers. Combination treatment of lapatinib plus capecitabine or letrozole had been used in patients with progressive human epidermal receptor 2 (HER2)-overexpressing metastatic breast cancer [13-15]. A phase III study demonstrated that the combination treatment of lapatinib and capecitabine significantly decreased the risk of disease progression compared to either drug alone [16]. However, an increased hepatobiliary disorders were reported in patients who received the combination treatment of lapatinib with paclitaxel, capecitabine or letrozole [17]. Other tyrosine kinase inhibitors including imatinib, nilotinib, dasatinib, gefitinib, erlotinib, sorafenib, sunitinib, pazopanib have also been reported to cause hepatotoxicity [18]. The underlying mechanisms of tyrosine kinase inhibitors-induced hepatotoxicity in combination treatment have not been fully elucidated.

In this study, we demonstrated that the increased incidence of hepatotoxicity during the combination treatment of lapatinib with paclitaxel was associated with an increased accumulation of the chemotherapeutic agents. Data from the *in vitro* and *in vivo* assays revealed that the increased drug accumulation was due to the inhibition of ABCB1 transport activity by lapatinib. A better understanding of the mechanism of this hepatotoxicity may facilitate the further optimization of the combination regimens composing lapatinib and other chemotherapeutic drugs in clinic.

## RESULTS

### A higher incidence of hepatotoxicity was observed in patients receiving combination therapy of lapatinib and paclitaxel than those receiving paclitaxel alone

From a cohort of 39 patients with HER2-overexpressing metastatic breast cancer, we analyzed the adverse events in hepatobiliary system from the start of the medication until 30 days after the last dose. No patients suffered any disease of the hepatobiliary system before receiving the chemotherapy. In the drug combination group (paclitaxel plus lapatinib), 4 patients experienced increased ALT and 2 patients experienced increased AST (Table 1). Most events were grade 1 or 2 according to the National Cancer Institute Common Terminology Criteria for Adverse Events (CTCAE) version 3.0. In addition, 4 patients who received the combination treatment showed hyperbilirubinaemia and one of them had a Grade 3 hyperbilirubinaemia. Furthermore, there was one patient in the group of lapatinib and paclitaxel withdrawn from the treatment because of severe abnormal hepatic function. In contrast, there were only 2 patients in the group of paclitaxel alone showed grade 1 abnormal hepatic function. Therefore, the clinical data showed that the combination therapy (paclitaxel plus lapatinib) group induced significantly more hepatotoxicity than the paclitaxel alone treatment group.

**Table 1:** Adverse events in the hepatobiliary system in a cohort of 39 patients

Group	ALT increased	AST increased	Hyperbilirubinaemia	Total
Paclitaxel alone	1/21 (4.76%)	1/21 (4.76%)	0/21 (0.00%)	2/21 (4.76%)
Paclitaxel + lapatinib	4/18 (22.22%)	2/18 (11.11%)	4/18 (22.22%) *	7/18 (38.89%) *

The data represent the number of subjects (%) in each group. The *P* value was calculated by Fisher's exact test.

* *P* < 0.05 for values versus those in paclitaxel alone.

### Lapatinib increased doxorubicin accumulation in ABCB1-overexpressing hepatocellular carcinoma cells *in vitro*

**Figure 1:**
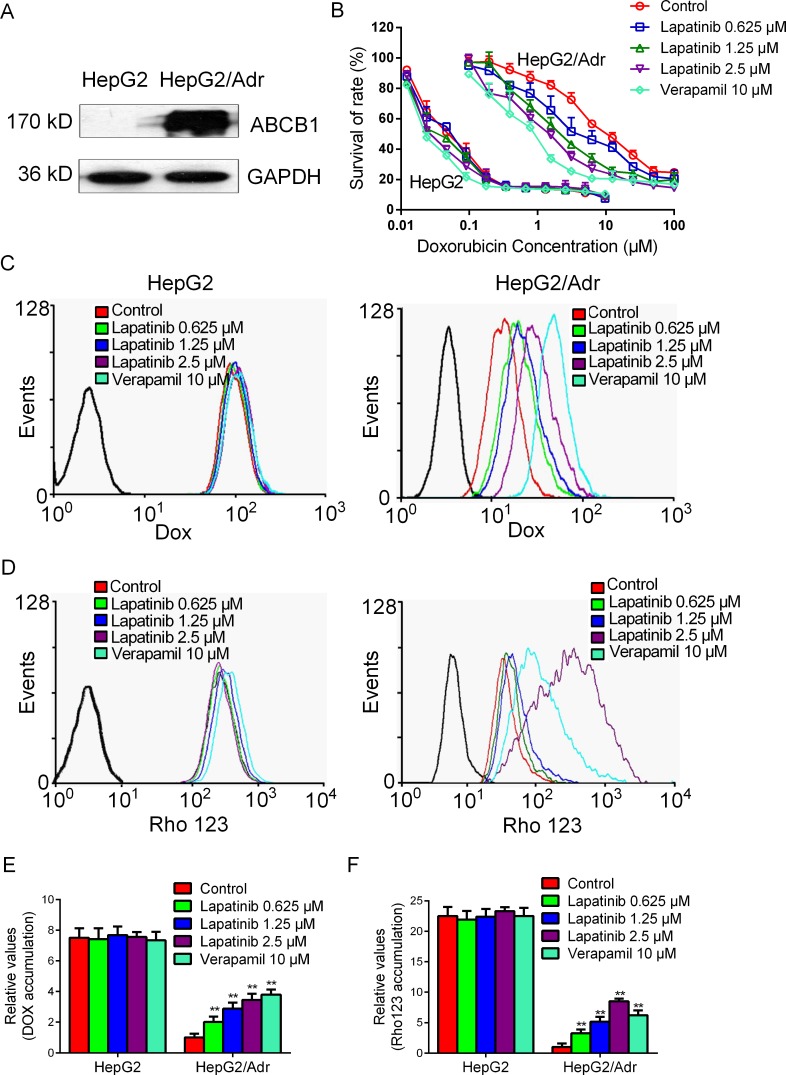
Effect of lapatinib on the accumulations of doxorubicin and rhodamine 123 **A.** The expression of ABCB1 in HepG2/Adr cells was detected by Western blot. **B.** Lapatinib enhanced the cytotoxicity effect of doxorubicin on ABCB1-overexpressing HepG2/Adr cells. **C. D. E. F.** The accumulations of doxorubicin and rhodamine 123 were measured by flow cytometric analysis as described in “Materials and Methods”. The results are presented as fold change in fluorescence intensity relative to control MDR cells. ** *P* < 0.01 versus control group. The experiment was done at least thrice.

We hypothesized that the increased hepatotoxicity was associated with elevated drug accumulation induced by lapatinib in the liver. In order to verify our conjecture, we first investigated the effect of lapatinib on increasing drug accumulation in a sensitive hepatocellular carcinoma cells HepG2 and its ABCB1-overexpressing resistant HepG2/Adr subline. The upregulation of ABCB1 expression in HepG2/Adr cells was shown as Figure 1A. Lapatinib was found to enhance the cytotoxicity effect of doxorubicin in HepG2/Adr cells in a concentration-dependent manner but not in the parental HepG2 cells (Figure 1B). Compared with the parental HepG2 cells, the intracellular accumulation of doxorubicin or an ABCB1 probe substrate rhodamine 123 in HepG2/Adr cells was decreased, suggesting that ABCB1 overexpression could reduce intracellular substrate accumulation (Figure 1). The fluorescent index of doxorubicin was increased by 1.85-, 2.15-, 3.10-fold in HepG2/Adr cell in the presence of 0.625, 1.25 and 2.5 μM of lapatinib, respectively (Figure 1C and 1E). Similarly, the fluorescent index of rhodamine 123 was increased by 2.23-, 3.21-, 9.77-fold in HepG2/Adr cell in the presence of 0.625, 1.25 and 2.5 μM of lapatinib, respectively (Figure 1D and 1F). In contrast, the intracellular accumulations of doxorubicin and rhodamine 123 were not altered in HepG2 cell in the presence of lapatinib.

**Figure 2:**
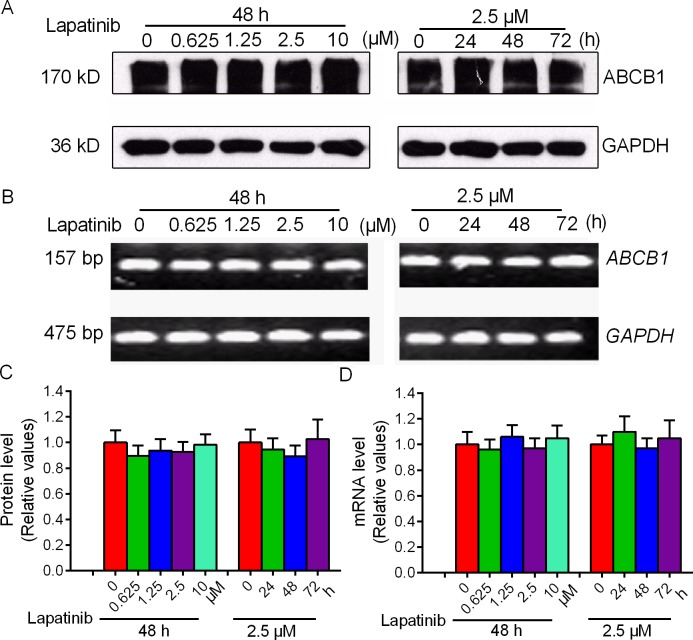
Effect of lapatinib on the expression of ABCB1 The protein level of ABCB1 was measured by Western blot and mRNA level was measured by qRT-PCR. **A. B.** Lapatinib did not alter the protein levels or mRNA levels in HepG2/Adr cells. **C.** Grayscale ratios of ABCB1/GAPDH were analyzed with Image J. The grayscale ratios were proportional to the ABCB1 protein levels. **D.** qRT-PCR was further applied to confirm unchangeable mRNA levels in HepG2/Adr cells. All experiments were repeated at least three times, and a representative set of data is shown in each panel.

Since the increased accumulation effect of lapatinib can be achieved either by antagonizing the drug efflux function of ABCB1 or by decreasing the expression level of ABCB1. We next examined the effect of lapatinib on the expression of ABCB1 at the mRNA and protein levels by qRT-PCR and Western blot analysis, respectively. As shown in Figure 2, ABCB1 RNA and protein expressions were not significantly altered in HepG2/Adr cells after treatment with lapatinib for 24, 48 or 72 h. These results suggested that lapatinib may increase intracellular accumulation of chemotherapeutic agents by inhibiting the function of ABCB1 without altering the transporter expression level.

### Lapatinib enhanced doxorubicin accumulation in normal liver tissues

**Figure 3:**
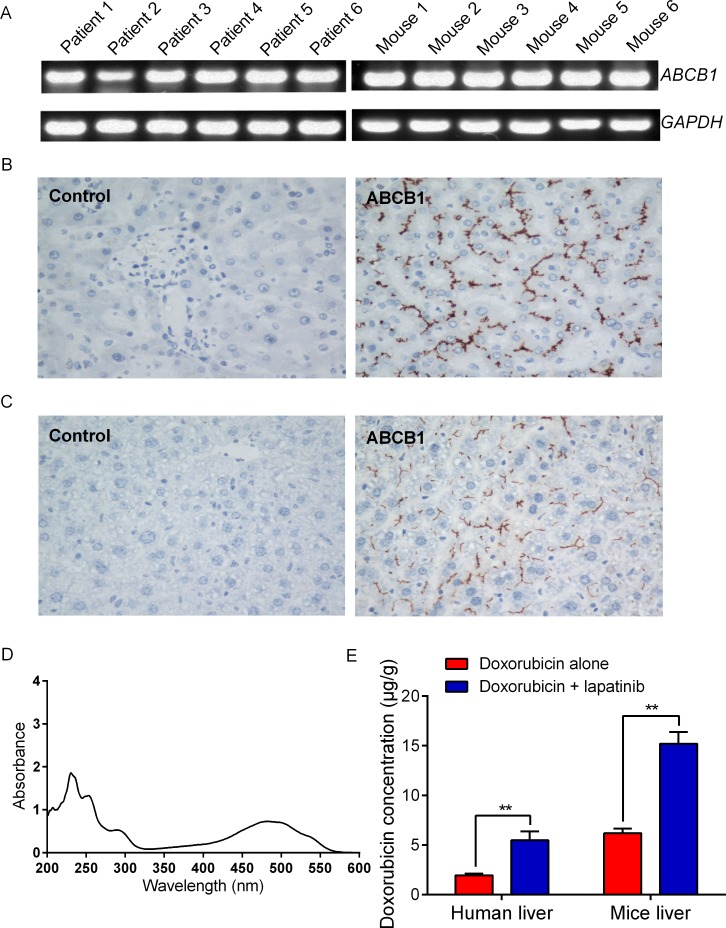
Expression levels of ABCB1 and doxorubicin accumulation in normal liver tissues The mRNA level was determined by qRT-PCR as described in “Materials and Methods”: **A.** the mRNA level of ABCB1 in normal liver tissues; **B.** Sections of human liver reacted with ABCB1 antibody, demonstrating localization in bile canaliculi and hepatocytes. **C.** Sections of mice liver reacted with ABCB1 antibody, demonstrating localization in bile canaliculi and hepatocytes; The magnification is 400 ×. **D.** The absorption spectra for doxorubicin (0.5 mg/ml) is detected by UV spectrophotometric method. **E.** lapatinib increased the doxorubicin accumulation in normal liver tissues. All experiments were done at least thrice.

To further understand the enhancement of chemotherapeutical agent mediated-hepatotoxicity by lapatinib, we investigated the effect of lapatinib on increasing drug accumulation in normal liver tissues obtained from Swiss mice and cancer patients. We first detected the expression levels of ABCB1 in human liver and mouse liver (6 cases for each group). The PCR results showed that normal human and mice liver both expressed high levels of ABCB1 (Figure 3A). Immunohistochemical staining confirmed that the normal liver specimens had high ABCB1 expression and that ABCB1 was localized on the biliary canalicular surface of hepatocytes and the apical surface of small biliary ductules (Figure 3B and 3C). Importantly, we found that there was much more accumulation of doxorubicin in human and mice liver tissues in the presence of lapatinib than in the absence of lapatinib (Figure 3E). The fluorescent index of doxorubicin was increased by 2.64-fold in human liver tissues when incubated in 2.5 μM lapatinib (Figure 3E). Similarly, the fluorescent index of doxorubicin was increased by 2.46-fold in Swiss mice liver tissues in the presence of 2.5 μM lapatinib (Figure 3E). Taken together, these results suggest that lapatinib could increase accumulation of ABCB1 substrate chemotherapeutical agents not only in ABCB1 overexpressing cancer cells but also in normal liver cells.

### Lapatinib altered the pharmacokinetics of paclitaxel and doxorubicin in mice

**Figure 4:**
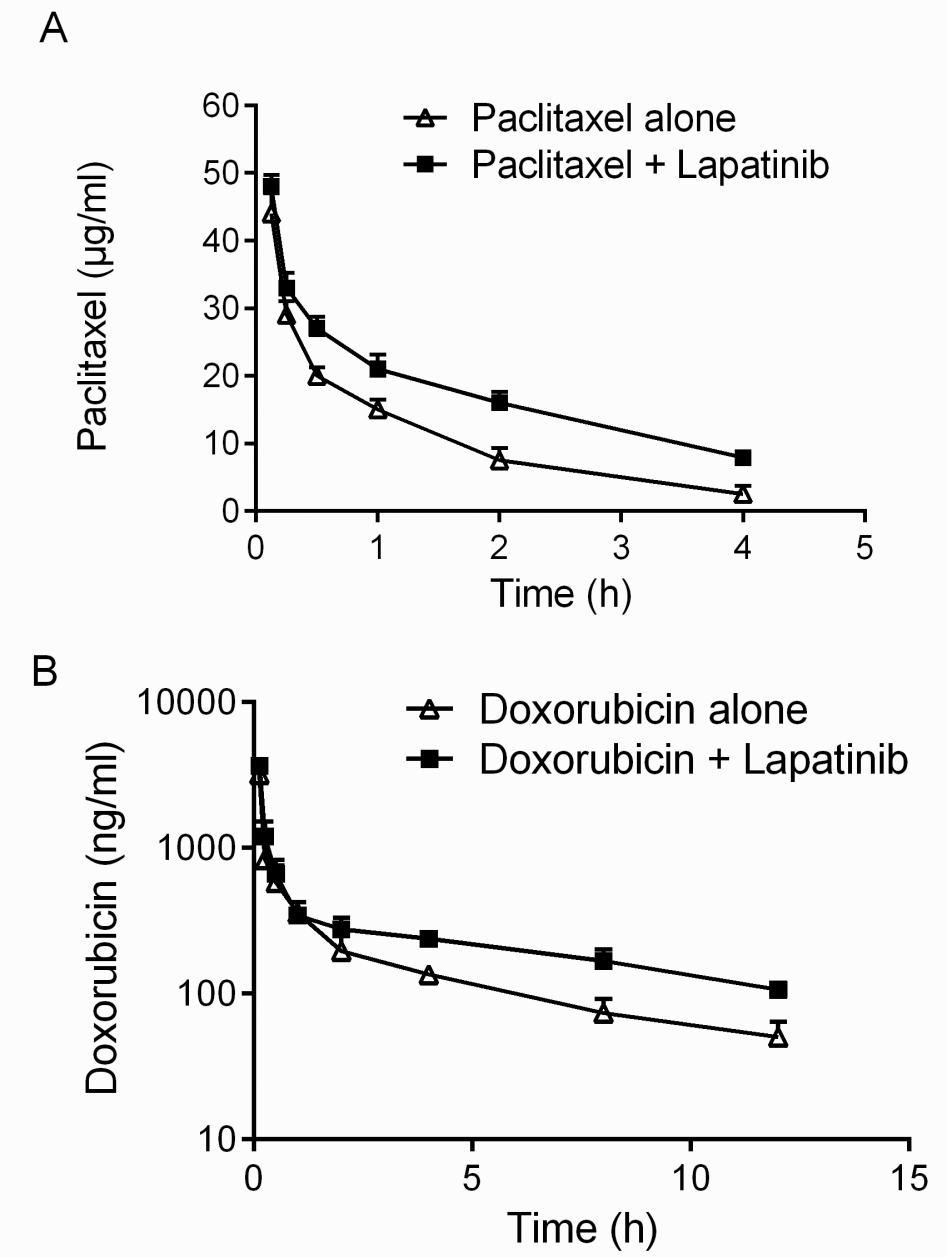
Effect of lapatinib on pharmacokinetics of paclitaxel and doxorubicin **A. B.** Effect of lapatinib on the profile of paclitaxel and doxorubicin pharmacokinetics in mice. NIH mice were p.o. administered with or without lapatinib (100 mg/kg) 1 h before i.v. administration of paclitaxel (18 mg/kg) or doxorubicin (10 mg/kg). HPLC analysis was performed as described in the “Material and Methods”. The values represent mean ± standard deviation in the triplicate determination.

Co-administration of lapatinib and an anticancer drug may significantly elevate plasma concentrations of anticancer drug by interfering with its clearance, which will increase the unpredictable side effect of the anticancer drugs [19, 20]. Since lapatinib has been shown to inhibit ABCB1, we examined the pharmacokinetic property of paclitaxel and doxorubicin in mice in the presence or absence of lapatinib. Our data indicated that the lapatinib plus paclitaxel combination group showed a significantly longer T_1/2_β and a greater area under concentration-time curve (AUC) than those in the paclitaxel alone group (Figure 4A, Table 2). Moreover, a significantly slower plasma clearance (CL) was observed in the paclitaxel plus lapatinib combination group (4.87 mL/h) than in the paclitaxel alone group (8.35 mL/h) (Table 2). Similar results were also observed for doxorubicin in the presence or absence of lapatinib (Figure 4B, Table 2). These findings indicated that lapatinib could increase the systematic exposure of ABCB1 substrate chemotherapeutic agents and may thereby increase the chance of adverse effect (e.g. hepatotoxicity) from the chemotherapeutic agents.

**Table 2:** The Pharmacokinetic parameters of paclitaxel and doxorubicin were summarized in the presence or absence of lapatinib

Group	T_1/2_β (h)	Vd (mL)	Clearance (mL/h)	AUC0→4 (h·ng/mL)	AUC0→∞ (h·ng/mL)	MRT (h)
Paclitaxel alone	1.24 ± 0.06	14.96 ± 1.28	8.35 ± 1.00	44.24 ± 4.62	49.26 ± 5.52	1.42 ± 0.08
Paclitaxel + lapatinib	1.88 ± 0.21 *	13.22 ± 1.34	4.87 ± 0.33 **	66.48 ± 4.04 **	84.50 ± 5.55 **	1.95 ± 0.10 **
Doxorubicin alone	5.20 ± 0.75	3.39 ± 1.21	3.57 ± 1.03	2921.15 ± 841.16	2956.42 ± 878.46	7.51 ± 1.08
Doxorubicin + lapatinib	15.50 ± 7.48 ^##^	2.28 ± 0. 64	1.95 ± 0.56 ^##^	5338.43 ± 1519.20 ^##^	5477.19 ± 1585.54 ^##^	22.36 ± 10.79 ^##^

The data represent the means and standard deviation of three independent samples and the *P* value was calculated by student *t* test. T_1/2_β = Elimination Half-life. Vd = Apparent volume of distribution. AUC = Area under plasma concentration/time curve. MRT = Mean residence time.

* *P* < 0.05

** *P* < 0.01 for values versus those in paclitaxel alone, respectively.

## *P* < 0.01 for values versus those in doxorubicin alone.

### Combination of lapatinib and paclitaxel/doxorubicin resulted in elevated hepatotoxicity *in vivo*

**Figure 5:**
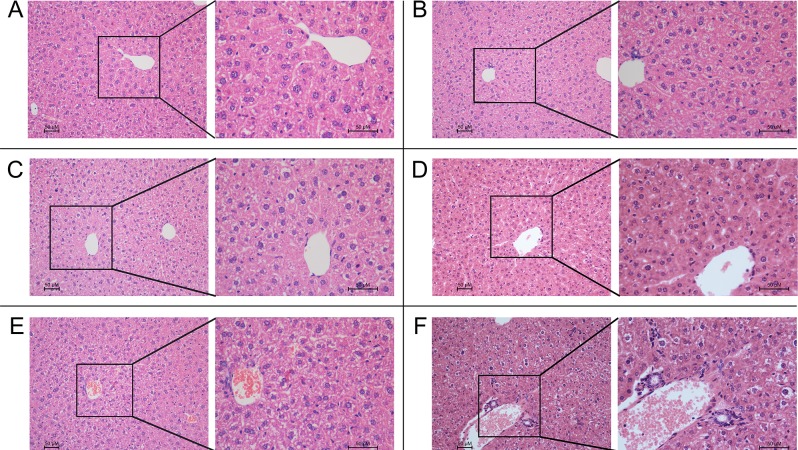
The histological results in *in vivo* experiments HE staining of liver tissues (Main-figure: magnification × 200; Sub-figure: magnification × 400) **A.** Normal liver parenchyma; **B.** (group of lapatinib alone) shows a similar histology to the normal group; **C.** (group of paclitaxel alone) shows mild liver damage: hepatocytes with hydropic changes and monocytes infiltration around the central vein; **D.** (group of doxorubicin alone) shows mild central venous hyperemia, Hepatic cord disorder and vacuolar degeneration; **E.** (group of lapatinib and paclitaxel) and **F.** (group of lapatinib and doxorubicin) show severe liver damage: severe inflammation with neutrophil and eosinophil in portal area, focal necrosis in lobular area, apoptotic body in periportal area. The values represent mean ± standard deviation in the triplicate determination.

The Swiss mice model was used to evaluate the potential hepatotoxic effect of the combination treatment of lapatinib with paclitaxel or doxorubicin. Result of various hepatic function markers were summarized in Table 3. The combination of lapatinib and paclitaxel was found to increase ALT (*P* < 0.05) and CRE (*P* < 0.05) more significantly than paclitaxel alone. Similarly, the combination of lapatinib and doxorubicin was found to elevate BUN and UA more remarkably than doxorubicin alone. Result from histopathological examination also revealed that the drug combination caused more severe cell necrosis and morphological damages than the chemotherapeutical agent alone (Figure 5). In the combination groups, morphological evidence of hepatocyte damage, including presence of parenchymal acinar transformation zones, vacuolization and hydropic degeneration of hepatocytes, were observed. Furthermore, extensive inflammation of the hepatocytes was also noted, as indicated by the presence of mononuclear cells besides neutrophil and eosinophil leucocytes in the portal areas. On the other hand, much milder morphological changes of the hepatocytes were observed in paclitaxel/doxorubicin alone group. Given the biochemical and histological results, we concluded that the combination therapy could produce a severe toxicity to liver.

## DISCUSSION

The combination of lapatinib and paclitaxel is an effective first-line therapy in patients with HER2-positive metastatic breast cancer [21, 22]. However, it has been reported that combination of lapatinib and paclitaxel or capectiabine was associated with a higher incidence of hepatotoxicity. Abnormal liver function tests were observed in patients receiving the combination regimens in a number of clinical trials, including one conducted in China with a high incidence of hyperbilirubinaemia (34.6%) [23]. Hepatic dysfunction is a leading cause of acute liver failure and it is the major reason for termination of medicines during their clinical development or early withdrawal following approval [24, 25]. The mechanisms causing the increased hepatotoxicity in combination therapy of lapatinib and paclitaxel are not well defined. In the present study, we first reported a potential mechanism of the increased hepatotoxicity via the inhibition of ABC transporters by lapatinib. Our study indicated that patients are more vulnerable to drug-induced hepatotoxicity upon treatment with combination of lapatinib and substrate drugs of ABC transporters such as paclitaxel and doxorubicin.

To illustrate the potential mechanism, we found that lapatinib remarkably increased the accumulation of doxorubicin and rhodamine 123 in ABCB1-overexpressing hepatocellular carcinoma cells and normal liver tissues in concentration-dependent manners. However, lapatinib did not significantly increase the drug accumulation in the parental sensitive cells to the anticancer agents. Since lapatinib did not alter the mRNA and protein expressions of ABCB1, the elevated drug accumulation of ABCB1 substrate chemotherapeutic drugs must be mediated by the inhibitory effect of lapatinib on ABCB1 transport activity [7, 8]. The *in vitro* drug accumulation assay and the pharmacokinetic study further confirmed that the increased hepatotoxicity by the drug combination was mediated by the increased drug accumulation induced by lapatinib. As a result, taking our data into account, the hepatoprotective effects could well be the result of inhibiting ABCB1 function. As our study showed, lapatinib would likely exacerbate the hepatotoxic effects of paclitaxel or doxorubicin rather than reduce them. Our findings have important implications for the clinical use of lapatinib in combination with ABCB1 substrate anticancer drugs. Doxorubicin and paclitaxel, which are commonly used in treating breast cancer, are all ABCB1 substrates. Possible drug-drug interactions with lapatinib may occur and cause severe hepatotoxicity. Therefore, evaluating genetic polymorphism of ABCB1 in patient before the commencement of treatment might be considered, especially when TKIs are included in the combination regimen, because the likelihood of hepatotoxicity may be predicted.

Combination chemotherapy of molecular targeted agents with other cytotoxic anticancer drugs has attracted a lot of attention recently. Early studies showed that gefitinib enhanced the anticancer activity of irinotecan by inhibiting ABCB1 and ABCG2 function, thereby increasing oral bioavailability and reducing clearance of topotecan in mice [26, 27]. Co-administration of lapatinib and irinotecan was also found to increase the area under the plasma concentration-time curve of SN-38, the active metabolite of irinotecan and ABCB1 substrate [28]. In a phase III trial of HER2-positive metastatic breast cancer patients whose cancers had not responded to trastuzumab or other therapies, superior therapeutic response were obtained by combination of lapatinib and capecitabine, compared with capecitabine alone [29]. Moreover, lapatinib given in combination with tamoxifen (substrate of ABCB1) was also found to effectively inhibit cell proliferation and restore tamoxifen sensitivity in ER-positive and tamoxifen-resistant breast cancer [30]. While lapatinib was also shown to provide clinical benefit when used in combination with other anticancer drugs in patients with brain metastasis breast cancer by promoting drug penetration through the blood brain barrier [31]. Penetration of taxanes and vinorelbine across the placenta was known to be regulated by ABCB1. By inhibiting ABCB1, co-administration lapatinib and paclitaxel has been shown to obscure the normal development of the fetal kidney and the regimen should be avoided during pregnancy [32]. We have previously demonstrated that lapatinib could reverse the ABC transporters-mediated MDR by inhibiting the efflux function of the transporters [7]. Therefore, the adverse effect from the lapatinib-containing combination regimens could be due to the inhibition of ABC transporters at important biological barriers. In fact, the pharmacokinetic drug-drug interactions related to the combination use of MDR modulators with other ABC transporter substrate drugs have been reported [33, 34], which necessitated dose reduction of the anticancer drugs in order to avoid adverse effects. Therefore, in this study, we deliberately set out to investigate the pharmacokinetics of paclitaxel and doxorubicin in the presence or absence of lapatinib. In human pharmacokinetic studies, the highest peak plasma lapatinib concentration was roughly 3 μM and the half-life was approximately 17 h with achievement of steady-state concentration after six to seven days of once-daily dosing [35, 36]. Therefore, the concentrations of lapatinib investigated in our *in vitro* experiments are clinically relevant. Our result proved that lapatinib significantly increased the exposure level of paclitaxel and doxorubicin in mice (Table 3). These suggest pharmacokinetics of conventional chemotherapeutic agent may be altered in the presence of lapatinib.

**Table 3:** Mean values of liver function tests in Swiss mice hepatotoxicity model

Group	ALT(U/L)	AST(U/L)	UA(mg/dL)	ALP(U/L)	LDH(U/L)	BUN(U/L)	CRE(μmol/L)	GLOB(mmol/L)
Control	28.22 ± 6.72	99.27 ± 11.44	286.4±64.6	218.51 ± 51.01	1542.05 ± 404.84	9.59 ± 1.14	17.87 ± 2.16	14.85 ± 0.25
Lapatinib	32.05 ± 5.57	99.59 ± 15.55	271.0±78.7	234.96 ± 54.33	1732.88 ± 489.41	10.95 ± 1.62	18.47 ± 2.19	14.1 ± 0.35
Paclitaxel	33.90 ± 6.09	97.94 ± 23.46	237.3±72.8	192.35 ± 32.12	1540.83 ± 457.35	9.82 ± 1.38	15.77 ± 1.78	14.12 ± 0.44
Paclitaxel + lapatinib	59.97 ± 26.72 *	107.10 ± 28.67	317.8±75.4	213.29 ± 53.45	1808.00 ± 610.33	10.23 ± 0.97	15.39 ± 1.85 *	17.95 ± 0.27
Doxorubicin	22.19±15.00	84.71±11.08	440.27±78.04 *	112.3±6.91	1606.32±505.09	7.91±0.82	20.17±7.14	18.74±1.83
Doxorubicin+ lapatinib	20.43±8.61	101.44±10.98	523.42±50.73 **	113.85±18.66	1691.27±623.47	8.00±0.90	20.6±5.50	20.80±1.97 *

The *P* value was calculated by student *t* test. The values represent mean ± standard deviation in the triplicate determination.

* *P* < 0.05

** *P* < 0.01 for values versus those in control.

In the *in vivo* animal study, the hepatotoxicity marker (ALT) was found to be much more remarkable upregulated after treatment with the drug combination (lapatinib and paclitaxel) than the individual drug alone. The observed increased serum ALT is almost always a consequence of release by dead or dying hepatocytes, and is a sensitive semi-quantitative measure of liver injury. Serum ALT elevations can identify hepatocyte injury or necrosis, which can be transient, as the liver adapts to repair toxic injury. This compensation action in the liver could explain why the ALT level in group of lapatinib and doxorubicin was not raised. From the above we can conclude that the treatment of lapatinib plus paclitaxel or doxorubicin could cause a severe liver injury than the single-agent treatment. Hepatotoxicity induced by lapatinib-containing combination was further confirmed by histopathological examination of liver tissue specimens. The histopathological evaluations demonstrated hydropic degeneration, vacuolated hepatocytes, portal inflammation and additionally apoptotic cells and lobular focal necrosis with extended exposure in the combination group of lapatinib and paclitaxel or doxorubicin. In addition, we observed a serious sinusoidal injury and hyperemia in the combination treatment group. These pathological changes are consistent with the cytotoxic effects induced by paclitaxel and doxorubicin. The morphology of the liver injury induced by doxorubicin is related to the hydropic degeneration, hemorrhage, heavy centrilobular spotting and extensive depletion in glycogen while the morphological changes in liver induced by paclitaxel are inflammatory cell infiltrates and foci, sinusoidal expansion, vesicular degeneration, and necrotic cells [37, 38]. However, all of these features were absent from livers of mice receiving monotherapy (paclitaxel or doxorubicin) treatment at the dose tested. These results indicated that lapatinib could exacerbate the liver injury induced by paclitaxel and doxorubicin. It has been reported that doxorubicin and paclitaxel could damage rat liver by inducing oxidative stress [39]. Further studies are needed to evaluate whether lapatinib could enhance the effects of paclitaxel and doxorubicin through promoting oxidative stress.

**Figure 6:**
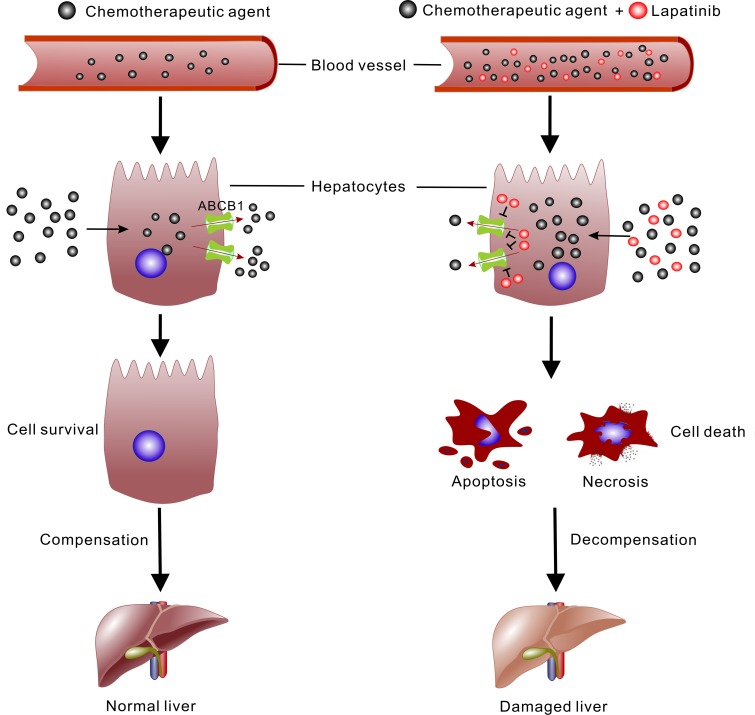
Schematic illustration of the mechanism of lapatinib promoting the cytotoxic effect of chemotherapeutic agent in liver Lapatinib can interfere with the distribution of agent and increase the plasma concentration. In hepatocytes, the chemotherapeutic agent is excreted out of the cells by ABC transporters (mainly ABCB1). Lapatinib inhibits the function of ABCB1 and elevates the concentration of chemotherapeutic agent in hepatocytes. Consequently, the hepatocytes develop into apoptosis or necrosis and the hepatic function is disordered.

In conclusion, the present study demonstrated for the first time the hepatotoxicity caused by the co-administration of lapatinib and paclitaxel/doxorubicin by both biochemical and histopathological evaluation. We showed that lapatinib can increase the accumulation of chemotherapeutic agents especially ABCB1 substrates in liver to give rise to the higher incidence of hepatotoxicity (Figure 6). Given that ABC transporters are also highly expressed in many normal organs such as liver, colon, kidney and brain, dose adjustment of the concomitantly administered chemotherapeutic drugs is needed to avoid adverse events including hepatotoxicity in combination regiment involving ABC transporter inhibitor.

## MATERIALS AND METHODS

### Chemicals and reagents

Doxorubicin (Dox), paclitaxel, verapamil, 3-(4,5-dimethylthiazol-yl)-2,5-diphenyllapatinibrazolium bromide (MTT) and rhodamine 123 (Rho 123) were purchased from Sigma Chemical Co. (St. Louis, MO, USA). Lapatinib was purchased from LC laboratories (Woburn, MA, USA). Dulbecco's modified Eagle's medium (DMEM) were products of Gibco BRL (Grand Island, NY, USA). Monoclonal antibodies against ABCB1 were purchased from Santa Cruz Biotechnology Inc (Santa Cruz, CA, USA). Other routine laboratory reagents were of analytical grade obtained from various commercial sources (Guangzhou, China).

### Cell lines and cell culture

The human hepatocellular carcinoma cell line HepG2 was obtained from American Type Culture Collection (ATCC). The Dox-selected derivative ABCB1-overexpressing HepG2/Adr subline was established as previous study [40]. All cells were grown in DMEM supplemented with 10% fetal bovine serum, 100 units/mL streptomycin sulfate and 100 units/mL penicillin G sulfate, and maintained at 37°C in the presence of 5% CO_2_. HepG2/Adr cells were cultured with 1.2 μM Dox to maintain its ABCB1-overexpressing properties [40]. All cells were grown in drug-free culture medium for more than 2 weeks before assay.

### Patients

The medical history of a total of 39 patients with HER2-overexpressing MBC (March 2006 - October 2008) were retrieved from the database archive of the Sun Yat-sen University (Guangzhou, China) for analysis. All patients were diagnosed with invasive breast cancer by histological examination. The HER2 overexpression was confirmed by fluorescence in situ hybridization. 18 patients received the combination treatment (lapatinib 1500 mg daily plus paclitaxel 80 mg/m^2^ every 3 days) and the control group received only paclitaxel (80 mg/m^2^ every 3 days). The median age is 50.5 years (age range from 21 to 65 years).

### Western blot analysis

The HepG2 and HepG2/Adr cells were exposed to different concentrations of lapatinib (0.625, 1.25, 2.5 and 10 μM) for different periods (0, 24, 48 and 72 h) to test whether lapatinib affected the expression of ABCB1. Western blot analysis was conducted as previously described [7]. After being incubated in blocking solution containing 5% non-fat milk in TBST buffer (10 mM Tris-HCL (pH 8.0), 150 mM NaCl, and 0.1% Tween 20) for 2 h at room temperature, membranes were incubated with primary antibody ABCB1 (1:1000 dilution, Santa Cruz Biotechnology) and GAPDH (1:5000 dilution, Kangcheng Biotechnology). The membranes were then incubated for 2 h with HRP-conjugated secondary antibody at 1:5000 dilution. Immunoreactive bands were visualized by the enhanced Phototope TM-HRP Detection Kit (Cell Signaling, USA) and exposed to Kodak medical X-ray processor (Carestream Health, USA). Protein expression was quantified by Scion Image software (Scion Co, USA).

### Cytotoxicity

We collected the cells and seeded them at a density of 3.0 × 10^3^ cells per well in 96-well plates. After 24 h, different concentrations of doxorubicin were added into the wells 1 h after lapatinib was added. After 68 h, MTT (5 mg/mL, 20 μL) was added into each well and cultured for another 4 h. Then the medium was discarded and each well was added with 120 μL DMSO. Finally, optical density was measured at 540 nm with background subtraction at 670 nm by Model 550 Microplate Reader (Bio-Rad, Hercules, CA, USA).

### Doxorubicin and rhodamine 123 accumulation

The accumulation of doxorubicin or rhodamine 123 in HepG2 and HepG2/Adr cells was measured as previously described [41]. Verapamil, a known ABCB1 inhibitor, was used as a positive control [42].

### *Ex vivo* evaluation of doxorubicin accumulation in patient liver tissues

The fresh normal liver tissue adjacent to the hepatocellular carcinoma retrieved from Sun Yat-sen university cancer center was used for the drug accumulation assay *ex vivo*. Informed consent was obtained from the patients before tissue collection. The work was approved by the ethics review committee at Sun Yat-sen University (Guangzhou, China). Liver tissues of 50-100 mg were cut into small pieces (1mm^3^) and cultured in DMEM containing 15% fetal bovine serum at 37°C. The tissues were incubated with the desired concentration of lapatinib at 37°C for 1 h in the medium, and then 10 μM doxorubicin was added to the medium and further incubated for 6 h. Subsequently, the tissues were then collected, centrifuged and washed twice with cold PBS containing 10 μM verapamil. Then, liver tissues were resuspended in 1 ml/100 mg tissues lysis solution which contained 0.3 mol/l HCl and 60% ethanol (1:1) and was homogenized at 4°C and then centrifuged at 12 000 × g [43]. The supernatant was removed and its fluorescence signal was measured by a spectrofluorometer at *λ_ex_* 470 nm and *λ_em_* 590 nm. Simultaneously, the standard curve of doxorubicin was made with the same condition. The doxorubicin accumulation in liver tissues (μg/g) was calculated according to the fluorescence value using a standard curve.

### Immunohistochemistry

Immunohistochemical staining for ABCB1 expression was performed using a standard two-step method [44]. The tissues were fixed with 4% paraformaldehyde in 0.1 M phosphate buffer for 24 h. After fixation, the tissues were embedded in paraffin wax. The paraffin tissues were sliced into 14 μM sections and mounted onto glass slides. After dewaxing, antigen retrieval was facilitated in Tris-EDTA buffer (pH 9.0) and the tissue sections were then incubated with primary antibodies against ABCB1 (1:100 dilution, Santa Cruz Biotechnology) for overnight at 4°C. Then the slides were washed three times with PBS and subsequently incubated with second antibodies and stained with 3,3-diaminobenzidine tetrahydrochloride (DAB). Finally, the slides were counter stained with Mayer's hematoxylin. Slides immunoreacted with only second antibodies provided the background staining for comparison. All slides were examined with a Nikon Eclipse 80i microscope and images were captured with NIS-Elements F 3.2 imaging analysis system.

### Swiss mice hepatotoxicity model

Swiss mice, 5 - 6 weeks of age and weighing about 20 g, were obtained from the Center of Experimental Animals, Sun Yat-sen University (Guangzhou, China) and were randomized into six treatment groups: (1) saline (on day 1 and 2 of every week for 2 weeks); (2) lapatinib alone (on day 1 and 2 of every week for 2 weeks, p.o., 100 mg/kg); (3) paclitaxel alone (on day 2 of every week for 2 weeks, i.v., 18 mg/kg); (4) paclitaxel (i.v., 18 mg/kg) one hour after lapatinib on day 2 of every week, (5) doxorubicin alone (every three days for 2 weeks, i.v., 10 mg/kg); (6) doxorubicin (i.v., 10 mg/kg) one hour after lapatinib (p.o., 100 mg/kg) every three days. The experimental animals had free access to sterilized food and water. Liver function was evaluated by measuring the levels of aspartate aminotransferase (AST), alanine aminotransferase (ALT), total bilirubin, albumin and alkaline phosphatase (ALP), lactate dehydrogenase (LDH), gamma glutamyl transferase (GGT). After 2 weeks, all mice were euthanized by cervical dislocation and unprocessed blood and tissues samples were quickly obtained from the mice. Liver tissues specimens were fixed in 10% formaldehyde and embedded in paraffin blocks. Sectioned slices were stained with haematoxylin and eosin (H&E), and each section was analyzed at 200 and 400·magnification by Nikon Eclipse 80i microscope. All of the pathological slides were independently and blindly evaluated by two pathologists (Dr Shixun Lu and Dr Rongzhen Luo) from the department of pathology of Sun Yat-sen University Cancer Center. All animal care and experimental procedures were approved by the Ethics Committee for Animal Experimentation and were carried out in accordance with the guidelines on animal care and experiments of laboratory animals (Center of Experimental Animals, Sun Yat-sen University, China).

### Pharmacokinetic study in NIH mice

Male and female NIH mice weighing 20-23 g were obtained from the Center of Experimental Animals, Sun Yat-sen University (Guangzhou, China) for the pharmacokinetic study. The mice were randomly divided into four groups (paclitaxel alone, doxorubicin alone and their combination with lapatinib), each of which consisted of 6 mice (three males and three females). For plasma paclitaxel levels, mice were treated with either 100 mg/kg of lapatinib (p.o.) or vehicle on days 1 and 2 and on day 2 all mice were injected with 18 mg/kg of paclitaxel (10 mL/kg) via the caudal vein one hour after lapatinib or vehicle. For plasma doxorubicin levels, mice were treated with either 100 mg/kg of lapatinib (p.o.) or vehicle on days 1 and 2 and on day 2 all mice were injected with 10 mg/kg of doxorubicin (10 mL/kg) via the caudal vein one hour after lapatinib or vehicle. Blood was collected from the retro-orbital plexus at designated time points and they were kept in cold heparin coated glass tubes. The blood from 1 male and 1 female was pooled and plasma was isolated and stored at −80°C until analysis. The plasma levels of paclitaxel and doxorubicin were analyzed by HPLC as previously described (20). Pharmacokinetic parameters was determined from the concentration-time data by non compartment model using the 3P97 software (Practical Pharmacokinetics Software, Beijing, China). All experiments were approved by Ethics Committee for Animal Experiments and carried out in accordance with the guidelines on Animal Care and Experiments of Laboratory Animals of Center of Experimental Animals, Sun Yat-sen University.

### Reverse transcription-PCR

Total cellular RNA was isolated by Trizol Reagent (Gibco BRL, USA) RNA extraction kit following manufacture instruction. cDNA were prepared from the sensitive HepG2 and the resistant HepG2/Adr cells. Reverse transcription was done with reverse transcriptase (Promega Corp., Madison, WI). Oligonucleotides primers for *ABCB1* and *GAPDH* were synthesized commercially (Invitrogen Co., China). They included *ABCB1* (homo sapiens), sense primer, 5′-CCCATCATTGCAATAGCAGG-3′, antisense primer, 5′-GTTCAAACTTCTGCTCCTGA-3′; *Abcb1* (mus), sense primer, 5′-CCCATGGCTGCATCAGTGTT-3′, antisense primer, 5′-GCTGAGTGCCTTTGTCTCCT-3′; *GAPDH*, sense primer, 5′-GAAGGTGAAGGTCGGAGTC-3′, antisense primer, 5′-GAAGATGGTGATGGGATTTC-3′. Regular PCR was performed at 94°C for 2 min for initial denaturation, and then at 94°C for 30 seconds, 56°C for 30 seconds, and 72°C for 1 min using the GeneAmp PCR system 9700 (PE Applied Biosystem, Foster City, CA). After 35 cycles of amplification, additional extensions were done at 72°C for 10 min. Products were resolved and examined by 1% agarose gel electrophoresis [45]. For qRT-PCR analysis, the reaction was carried out at 50°C for 2 min, 95°C for 5 min and 40 cycles at 95°C for 15 s, 60°C for 30 s. Relative quantification of *ABCB1* was performed using the 2^−ΔΔCt^ method [46]. All experiments were repeated three times.

### Statistical analysis

The Fisher's exact test was used to evaluate the statistical significance in clinical data. In all other experiments, statistical significance was determined at *P* < 0.01 (**) or *P* < 0.05 (*) by the Student's *t*-test. The data was expressed as means ± standard deviation.
